# Swarming Insects May Have Finely Tuned Characteristic Reynolds Numbers

**DOI:** 10.3390/biomimetics9110660

**Published:** 2024-10-30

**Authors:** Andy Reynolds

**Affiliations:** Rothamsted Research, Harpenden, Hertfordshire AL5 2JQ, UK; andy.reynolds@rothamsted.ac.uk; Tel.: +44-(0)1582-763133

**Keywords:** collective motion, swarming, stochastic modelling, turbulence, Reynolds numbers

## Abstract

Over the last few years, there has been much effort put into the development and validation of stochastic models of the trajectories of swarming insects. These models typically assume that the positions and velocities of swarming insects can be represented by continuous jointly Markovian processes. These models are first-order autoregressive processes. In more sophisticated models, second-order autoregressive processes, the positions, velocities, and accelerations of swarming insects are collectively Markovian. Although it is mathematically conceivable that this hierarchy of stochastic models could be extended to higher orders, here I show that such a procedure would not be well-based biologically because some terms in these models represent processes that have the potential to destabilize insect flight dynamics. This prediction is supported by an analysis of pre-existing data for laboratory swarms of the non-biting midge *Chironomus riparius*. I suggest that the Reynolds number is a finely tuned property of swarming, as swarms may disintegrate at both sufficiently low and sufficiently high Reynolds numbers.

## 1. Introduction

Mating swarms of flying insects are a form of collective animal behaviour that typically displays a high degree of spatial cohesion but lacks the ordered collective movements that are a defining feature of herds, flocks, and schools [1,2,3,4,5]. As the insects circulate within the swarm, they execute erratic flight patterns [1,2,6,7]. Over the last few years, there has been much effort put into the development and validation of stochastic models of the trajectories of swarming insects [8,9,10,11,12]. These models account for numerous observations, including the emergence of dynamical scaling and correlations in perturbed swarms, the emergence of macroscopic mechanical properties like tensile strength, and the ability of swarms to be driven through ‘thermodynamic cycles’ by the application of external perturbations [13,14,15,16,17,18]. These models typically assume that the positions and velocities of swarming insects can be represented by continuous jointly Markovian processes, or more rarely, that the positions, velocities, and accelerations of swarming insects are collectively Markovian. Mathematically, these models can be seen to be the lowest levels in a hierarchy that could be extended to higher orders. Physically, the hierarchy corresponds to the inclusion of a timescale representative of the largest scales of motion at first order, and to the addition of a second timescale representative of the smallest scales of motion at second order. This is directly analogous to stochastic models of the trajectories of tracer particles in high Reynolds number turbulence, wherein the Reynolds number, $R={\left(\frac{T}{t_{2}}\right)}^{2}$, which is determined by the ratio of a timescale representative of the energy-containing scales, *T*, and the Kolmogorov time scale, $t_{2}$, representative of the dissipative scales of motions appears as a parameter at second order [19]. This Lagrangian Reynolds number is proportional to the better-known Eulerian Reynolds number [19]. Although it is mathematically conceivable that this hierarchy of stochastic models could be extended to higher orders [20,21,22], in the case of high Reynolds number turbulence is it not apparent that such a procedure would be well-based physically since there is no obvious relevant timescale smaller than the Kolmogorov timescale [19]. Here in the case of swarming insects, I show that the procedure is not well-based biologically.

## 2. Materials and Methods

Third-order one-dimensional models for the positions, x, velocities, u, accelerations, A, and jerks, J, of swarming insects are given by

(1) $$dJ=a\left(J,A,u,x\right)dt+bdW\left(t\right)$$

dA=Jdt

du=Adt

dx=udt

where $a\left(J,A,u,x\right)$ is featured in the Fokker–Planck equation:

(2) $$\frac{\partial P_{3}}{\partial t}+u\frac{\partial P_{3}}{\partial x}+A\frac{\partial P_{3}}{\partial u}+J\frac{\partial P_{3}}{\partial A}=-\frac{\partial }{\partial J}\left(aP_{3}\right)+\frac{b^{2}}{2}\frac{\partial^{2}P_{3}}{\partial J^{2}},$$

$P_{3}\left(J,A,u,x,t\right)$ is the joint distribution of J, A, u, x and time, t, b is the magnitude of the driving noise, and $dW_{i}\left(t\right)$ is an incremental Wiener process with correlation property $\bar{dW\left(t\right)dW\left(t+\tau \right)}=\delta \left(\tau \right)dt$ [20]. The prescription of $a\left(J,A,u,x\right)$ guarantees that the statistical properties of the simulated trajectories are distributed according to $P_{3}\left(J,A,u,x,t\right),$ which a model input. The deterministic term $a\left(J,A,u,x\right)$ takes the form:

(3) $$a=\frac{b^{2}}{2}\frac{\partial }{\partial J}lnJ+\frac{\phi }{P_{3}}$$

where

(4) $$\frac{\partial \phi }{\partial J}=\frac{\partial P_{3}}{\partial t}+u\frac{\partial P_{3}}{\partial x}+A\frac{\partial P_{3}}{\partial u}+J\frac{\partial P_{3}}{\partial A}$$

Integrating Equation (2) over all J gives an equation for the average jerk strength:

(5) $$0=\frac{\partial P_{2}}{\partial t}+u\frac{\partial P_{2}}{\partial x}+A\frac{\partial P_{2}}{\partial u}+\langle J\rangle \frac{\partial P_{2}}{\partial A}$$

where $P_{2}\left(A,u,x,t\right)$ is the joint distribution of A, u, x and time, t.

Integrating Equation (6) over all J gives an equation for the average acceleration:

(6) $$0=\frac{\partial P_{1}}{\partial t}+u\frac{\partial P_{1}}{\partial x}+\langle A\rangle \frac{\partial P_{1}}{\partial u}$$

where $P_{1}\left(u,x,t\right)$ is the joint distribution of u, x and time, t.

The least biased choice for $P_{3}\left(J,A,u,x,t\right)$ and the one adopted here is a multivariant Gaussian. The resulting stochastic models for the trajectories of swarming insects are minimally structured (maximum entropy) models. It follows from Equations (3)–(6) that for stationary swarms with Gaussian statistics:

(7) $$\langle A\rangle =-\frac{\sigma_{u}^{2}}{\sigma_{x}^{2}}x$$

(8) $$\langle J\rangle =-\left(\frac{\sigma_{A}^{2}}{\sigma_{u}^{2}}+\frac{\sigma_{u}^{2}}{\sigma_{x}^{2}}\right)u$$

(9) $$\langle S\rangle \equiv \frac{\phi }{P_{3}}=-\left(\frac{\sigma_{J}^{2}}{\sigma_{A}^{2}}+\frac{\sigma_{A}^{2}}{\sigma_{u}^{2}}+\frac{\sigma_{u}^{2}}{\sigma_{x}^{2}}\right)A-\frac{\sigma_{J}^{2}}{\sigma_{A}^{2}}\frac{\sigma_{u}^{2}}{\sigma_{x}^{2}}x$$

Insect trajectories were simulated by numerically integrating the stochastic models. Other predicted quantities, such as velocity spectra, were obtained analytically, as detailed in the Appendix A, Appendix B and Appendix C. A full description of the experimental setup that provided data for the model validation can be found in Sinhuber et al. [23].

## 3. Results

The predicted average acceleration towards the centre is a defining feature of insect swarms that keeps the swarms intact [2]. As observed, this effective force increases linearly as the distance from the swarm centre increases. Individuals in real and simulated swarms therefore behave on average as if they are trapped in elastic potential wells. Model predictions, Equation (8), for the average strength of the jerks, are in quantitative agreement with observations of asymptotically large swarms [5] containing on average 15 to 94 individuals [24]. This correspondence indicates that swarming insects are described by second or higher-order models.

The average strength of the snaps, 〈S〉, is seen to enter the model formulation at third order. One contribution to this quantity is aligned with the acceleration vector, which itself tends to be aligned with the position vector (Equation (7)). The other contribution to 〈S〉 is manifestly aligned with the position vector. This contrasts with the average strength of the jerks, which enter the model at second order and are aligned with the velocity vector, i.e., aligned with the direction of travel and so aligned with the major axis of the insect. Such alignment minimizes the impact that jerks can have on flight dynamics. This is not the case with snaps, which can momentarily be aligned with the minor axis of the insect, thereby maximizing their disruptive impact on flight dynamics. This suggests that swarming insects are at most described by second-order models.

**Figure 1:**
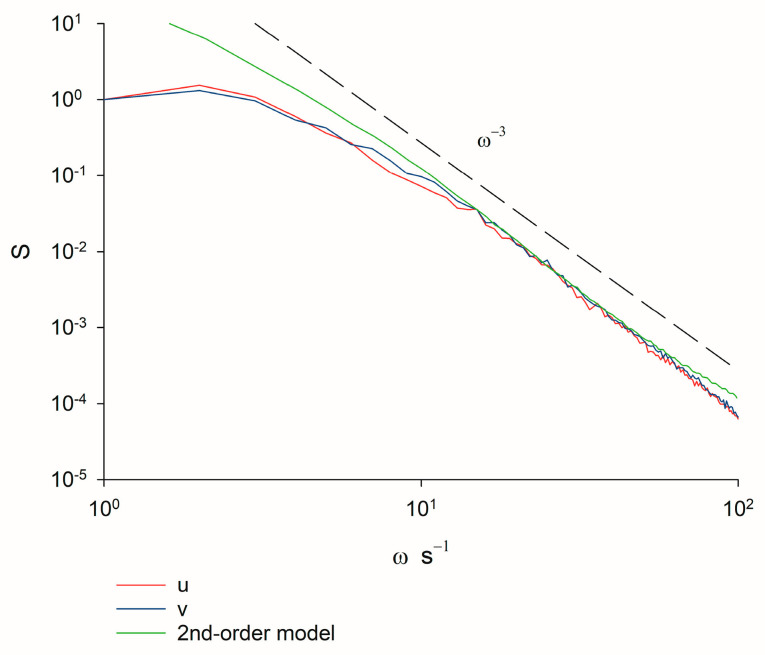
Velocity spectra, S, as a function of frequency, ω, obtained by analysis of pre-existing data for laboratory swarms of the non-biting midge Chironomus riparius. Results are shown for the largest swarm in the dataset of Sinhuber et al. [23], which on average contains 94 individuals. Results are shown for the two horizontal components of velocity. The spectra are seen to decrease as $\omega^{-3}$ at the highest frequencies accessible in the experiment. Recordings were made at a rate of 100 Hz. The same scaling behaviour was obtained for smaller swarms. Shown for comparison is the velocity spectra predicted by a 2nd-order model with T= $1,\;t_{2}=0.1$, $\sigma_{x}^{2}=1$ and $\sigma_{u}^{2}=1$ a.u. (Appendix A).

Experimental support for this prediction hinges on the fact that the velocity spectra for swarming insects are compatible with predictions from second-order models. Free-roaming trajectories are predicted by first- and second-order models to be characterised by velocity spectra that decrease respectively as $\omega^{-2}$ and as $\omega^{-4}\;$at high frequencies, whereas spectra decreasing faster than $\omega^{-4}\;$can only be captured by third- or higher-order models [19,20]. Confinement within a swarm does not change these scaling behaviours (Appendix A [25]). Instead, the quantity $\frac{\sigma_{u}}{\sigma_{x}}$ determines the position of the peak in the velocity spectra. The velocity spectra characterising the trajectories of swarming non-biting midge Chironomus riparius recorded in quiescent conditions in the laboratory decreases approximately as $\omega^{-3}$ at the highest frequencies accessible (Figure 1). This scaling cannot arise in first-order models but, as illustrated, does arise at low frequencies in second-order models when $R\sim O\left(100\right)$. This scaling is obtained for 19 different containing on average 15 to 94 individuals. Third-order processes are not evident.

## 4. Discussion

Herein it was argued that the trajectories of swarming insects, like the trajectories of tracer particles in turbulence, are at most described by second-order models in which the position, velocity, and acceleration of an insect are collectively Markovian, since higher-order processes, even if present, are not significant. This strengthens previously identified correspondences between swarming insects and the Lagrangian properties of high Reynolds number turbulence [24]. Their acceleration statistics have similar conditional dependencies on velocity. These conditional dependencies only become apparent for $\left|u\right|>2\sigma_{u},$ and their occurrences may be attributed to occasional energetic rotations. The small size of this Reynolds number (as determined by matching predicted and observed velocity spectra), $R\sim O\left(100\right)$, may be a consequence of the fact that the average strength of the jerks, Equation (8), increases with increasing Reynolds number. Equation (8) can be rewritten as $\langle J\rangle =-\left(\frac{R^{1/2}}{T^{2}}+\frac{\sigma_{u}^{2}}{\sigma_{x}^{2}}\right)u$. At sufficiently high Reynolds numbers, jerks, like snaps, may have the potential to destabilize flight dynamics, thereby causing the swarm to disintegrate. Indeed, the smallness of the estimate for the Reynolds number may be indicative of the susceptibility to swarming midges to the disruptive impact of jerks. The swarm may also disintegrate at sufficiently low Reynolds numbers following a disordered-order phase transition [12] if the confining potential, a collective emergent property of disordered swarms [1,2], cannot emerge in ordered swarms, or it may lose its collective properties if individuals remain in the vicinity of the swarm marker (a visually prominent marker over which swarm form). If this line of reasoning is correct, then the Reynolds number may be the result of fine-tuning, as are other emergent properties of swarming [12,26]. Jerks may also be particularly disruptive in swarms that are not asymptotically large (Appendix B [27,28]).

To summarise, even though the collective animal motions can exhibit fluidic behaviours, Reynolds numbers, which are perhaps the most ubiquitous quantity in the literature on fluid dynamics, have barely been featured in the literature on collective animal behaviours [29]. Herein, I addressed this shortcoming by showing that the trajectories of swarming insects are analogous to the trajectories of tracer particles in fluidic turbulence and that consequently swarming insects can be assigned Reynolds numbers. This new result strengthens ongoing attempts to describe collective animal behaviours as active matter [30] Moreover, the Reynolds numbers were shown to convey important new biological information, as all theoretical advances into collective animal behaviours should strive to do [31].

## Figures and Tables

**Figure A1:**
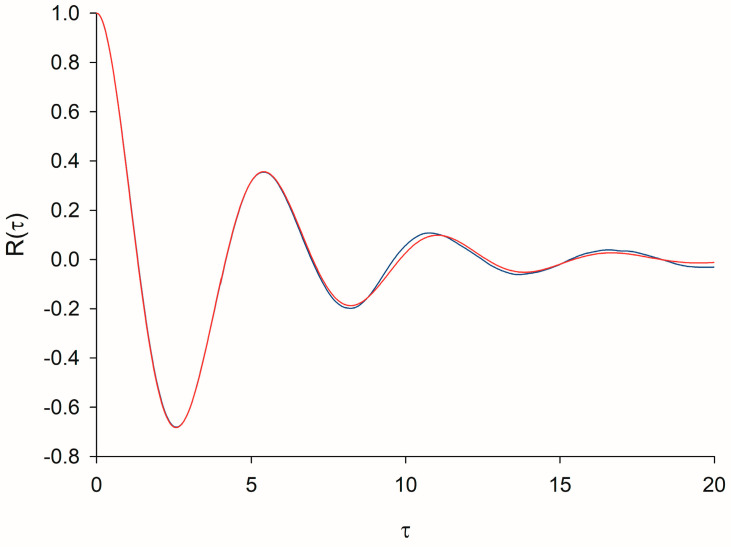
Predicted form of the velocity autocorrelation (red), Equation (A7), $R\left(\tau \right)$, matches that obtained in numerical simulations (blue). The trajectories of 10,000 swarming insects were simulated by numerically integrating Equation (A4). Results are shown for T = $t_{2}=1$, $\frac{\sigma_{u}^{2}}{\sigma_{x}^{2}}=1$ a.u.

**Figure A2:**
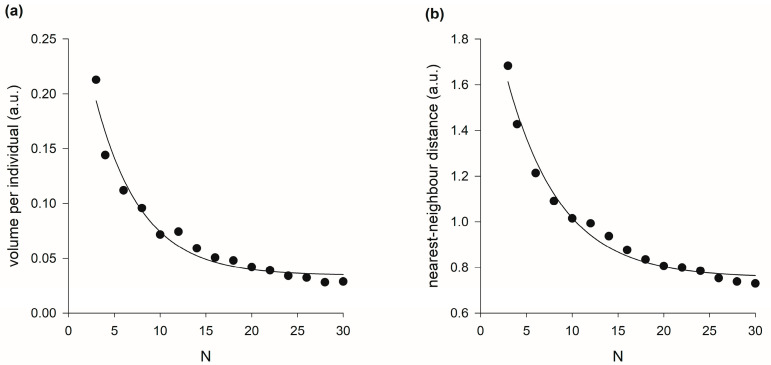
(**a**) Predicted volume per individual as a function of the number of individuals in the swarm. The solid line is an exponential fit, $V_{ind}=Aexp\left(-\frac{N}{N_{0}}\right)+B$ with $N_{0}\approx 5$. Puckett and Ouellette [5] reported that $N_{0}=3.1\pm 0.8$. (**b**) Predicted average distance from a given individual to its nearest neighbour as a function of the number of individuals in the swarm. The solid line is an exponential fit, $d_{nn}=Aexp\left(-\frac{N}{N_{0}}\right)+B$ with $N_{0}\approx 6$. Puckett and Ouellette [5] reported that $N_{0}=8.6\pm 2.0$. Predictions were obtained using the stochastic model of Reynolds [26].

**Figure A3:**
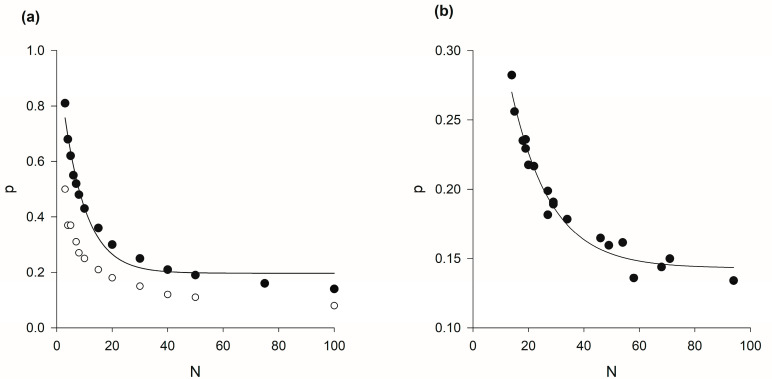
(**a**) Predicted and (**b**) observed net polarisations of acceleration as a function of the number of individuals in the swarm (●). The polarization $p=\langle \left|\overset{N}{\underset{i=1}{\sum }}\frac{A_{i}}{\left|A_{i}\right|}\right|\rangle /N$ where $A_{i}$ is the acceleration of the *i*th individual and where the angular brackets denote a time average. Predictions were obtained using the stochastic model of Reynolds [26]. The experimental data is taken from Sinhuber et al. [23]. The solid lines are exponential fits, $p=Aexp\left(-\frac{N}{N_{0}}\right)+B$ with (**a**) $N_{0}\approx 8$ and (**b**) $N_{0}\approx 14$. The average polarisations are significantly larger than the average polarisations that are predicted to arise sporadically in swarms of individuals with uncorrelated positions (o).

## Data Availability

Data sharing is not applicable to this article as no datasets were generated during the study.

## Appendix A

Power spectra for first- and second-order autoregressive models of swarming insects

Here I show that the power spectra predicted by first- and second-order autoregressive models of the trajectories of swarming insects decrease respectively as $\omega^{-2}$ and $\omega^{-4}$ at high frequencies. That is, I show that confinement within a swarm does not upset the scaling behaviours obtained by Sawford [19] for freely roaming trajectories.

First-order autoregressive models of swarming insects

Okubo [1] classic one-dimensional model for the positions, x, and velocities, u, of swarming insects is given by:

(A1) $$du=-\frac{u}{T}dt-\frac{\sigma_{u}^{2}}{\sigma_{x}^{2}}xdt+\sqrt{\frac{2\sigma_{u}^{2}}{T}}dW$$

dx=udt

where *T* is a velocity correlation timescale, $\sigma_{x}^{2}$ is the position variance, $\sigma_{u}^{2}$ is the velocity variance, and $dW_{i}\left(t\right)$ is an incremental Wiener process with correlation property $\bar{dW\left(t\right)dW\left(t+\tau \right)}=\delta \left(\tau \right)dt$.

Okubo [1] showed that according to this model the velocity autocorrelation function is given by:

(A2) $$R\left(\tau \right)=e^{-\frac{t}{2\tau }}\left(cos\omega_{1}\tau -\frac{1}{2\omega \tau }sin\omega_{1}\tau \right)$$

where $\omega_{1}={\left(\frac{\sigma_{u}^{2}}{\sigma_{x}^{2}}-\frac{1}{4T^{2}}\right)}^{1/2}$.

It follows that the velocity power spectra is given by:

(A3) $$f\left(\omega \right)=\int_{0}^{\infty }R\left(\tau \right)cos\omega \tau d\tau =\frac{\omega^{2}/T}{\left(\frac{1}{4T^{2}}+{\left(\omega_{1}-\omega \right)}^{2}\right)\left(\frac{1}{4T^{2}}+{\left(\omega_{1}+\omega \right)}^{2}\right)}$$

At sufficiently high frequencies $f\left(\omega \right)\propto \omega^{-2}$.

Second-order autoregressive models of swarming insects

The simplest second-order one-dimensional model for the positions, x, velocities, u, and accelerations, A, of swarming insects is given by:

(A4) $$dA=-\left(\frac{1}{T}+\frac{1}{t_{2}}\right)\left(A+\frac{\sigma_{u}^{2}}{\sigma_{x}^{2}}x\;\right)dt-\left(\frac{\sigma_{A}^{2}}{\sigma_{u\;}^{2}}+\frac{\sigma_{u}^{2}}{\sigma_{x\;}^{2}}\right)udt+\sqrt{2\sigma_{A\;}^{2}\left(\frac{1}{T}+\frac{1}{t_{2}}\right)\;}dW\;$$

du=Adt

dx=udt

where$\;\sigma_{A}^{2}=\sigma_{u}^{2}/Tt_{2}$ is the acceleration variance and $t_{2}$ is a timescale representative of the smallest scales of motion [25].

It follows that for this model, the velocity autocorrelation is a solution of the equation:

(A5) $$\frac{d^{3}R}{dt^{3}}+\left(\frac{1}{T}+\frac{1}{t_{2}}\right)\frac{d^{2}R}{dt^{2}}+\left(\frac{\sigma_{A}^{2}}{\sigma_{u\;}^{2}}+\frac{\sigma_{u}^{2}}{\sigma_{x\;}^{2}}\right)\;\frac{dR}{dt}+\left(\frac{1}{T}+\frac{1}{t_{2}}\right)\frac{\sigma_{u}^{2}}{\sigma_{x}^{2}}R=0$$

The simplest solutions are single exponentials $\;e^{-\xi \tau }$ where the inverse timescales ξ are solutions of the equation:

(A6) $$\xi^{3}-\left(\frac{1}{T}+\frac{1}{t_{2}}\right)\xi^{2}+\left(\frac{\sigma_{A}^{2}}{\sigma_{u\;}^{2}}+\frac{\sigma_{u}^{2}}{\sigma_{x\;}^{2}}\right)\xi -\left(\frac{1}{T}+\frac{1}{t_{2}}\right)\frac{\sigma_{u}^{2}}{\sigma_{x}^{2}}=0$$

Typically, one solution, $\xi_{1}$, is real whilst the other two, $\xi_{2}\pm i\Omega$, are a pair of complex conjugates. In this case, the general solution to Equation (A5) therefore takes the form:

(A7) $$R\left(\tau \right)=A\;e^{-\xi_{1}\tau }+\left(1-A\right)\;e^{-\xi_{2}\tau }\left(cos\Omega \tau -Bsin\Omega \tau \right)$$

where *A* and *B* are weighting factors.

It follows that:

(A8) $$f\left(\omega \right)=A\frac{\xi_{1}}{\xi_{1}^{2}+\omega^{2}}+\frac{1}{2}\left(1-A\right)\left(\left(\frac{\xi_{2}}{\xi_{2}^{2}+{\left(\Omega -\omega \right)}^{2}}+\frac{\xi_{2}}{\xi_{2}^{2}+{\left(\Omega +\omega \right)}^{2}}\right)+B\left(\frac{\xi_{2}-\Omega }{\xi_{2}^{2}+{\left(\Omega -\omega \right)}^{2}}+\frac{\xi_{2}+\Omega }{\xi_{2}^{2}+{\left(\Omega +\omega \right)}^{2}}\right)\right)$$

At sufficiently high frequencies:

$$f\left(\omega \right)\to c_{2}\omega^{-2}+c_{4}\omega^{-4}+O\left(\omega^{-6}\right)$$

where $c_{2}=A\;\xi_{1}+\left(1-A\right)\xi_{2}+\left(1-A\right)B\Omega$ and$\;c_{4}=\left(A-\left(1-A\right)B\right)\left(3\xi_{2}^{2}-\Omega^{3}\right)$

Because of the complexity of the general analytic expressions for ξ in terms of the model parameters, T, $t_{2}$, and $\sigma_{u}^{2}/\sigma_{u}^{2}$ precludes their application, here I present illustrative examples.

For T= $t_{2}=1$, $\frac{\sigma_{u}^{2}}{\sigma_{u}^{2}}=1$, $\xi_{1}\approx 1.54$, $\xi_{2,3}\approx 0.23\pm 1.12i$. The weighting factors *A* and *B* were found by minimizing the mean square difference between the predicted form of velocity autocorrelation, Equation (A7), and form obtained from simulation data obtained using Equation (A4) (Figure A1). This gave A=−0.24 and B=0.065. Consequently, $c_{2}≅0$ and $f\left(\omega \right)\propto \omega^{-4}$. The same scaling was obtained for T = $t_{2}=1$, $\frac{\sigma_{u}^{2}}{\sigma_{u}^{2}}=1/2$ and for T= $t_{2}=1$, $\frac{\sigma_{u}^{2}}{\sigma_{u}^{2}}=2$.

## Appendix B

Jerks may destabilize small swarms

Reynolds [26] showed that the positions of the non-biting midge *Chironomus riparius* in laboratory swarms are maximally anticorrelated. In this case, the average strength of the jerk experienced by the *i*th midge is given by:

(A9) $$\langle J_{i}\rangle =-\left(\frac{\sigma_{A}^{2}}{\sigma_{u}^{2}}\delta_{ij}+\frac{\sigma_{u}^{2}}{\sigma_{x}^{2}}\Lambda_{ij}\right)u_{j}$$

where $\Lambda_{ij}$ are elements of the inverse of the normalized position covariance matrix, the subscripts denote different individuals, and where there is a summation of over repeated indices. The average strength of the jerk experienced by the *i*th midge therefore depends on the velocities of all individuals in the swarm, and so is unlikely to be aligned with the direction of travel (the major axis of the insect). If it were aligned, then the impact that jerks can have on flight dynamics would be minimized (see main text). This misalignment is, however, small in asymptotically large swarms, which on average contain 10 or more individuals [5]. This is because $\langle \left|u\right|\rangle =\sqrt{\frac{2}{\pi }}\sigma_{u}N^{1/2}$ and because $\Lambda_{ij}=1$ when i=j and $\Lambda_{ij}=1/N$ when i≠j. Consequently,

(A10) $$\langle J_{i}\rangle =-\left(\frac{\sigma_{A}^{2}}{\sigma_{u}^{2}}+\frac{\sigma_{u}^{2}}{\sigma_{x}^{2}}\right)u_{i}-\frac{\sigma_{u}^{3}}{\sigma_{x}^{2}}O\left(N^{-\frac{1}{2}}\right)$$

Despite their disruptive influence in small swarms, the maximal positional anticorrelations appear to dictate the approach to the asymptotic state (Appendix C).

## Appendix C

Maximal anticorrelated positions and the asymptotic regime

Puckett and Ouellette [5] reported that once swarms contain order 10 individuals, all statistics saturate, and that the swarms enter an asymptotic regime. Puckett and Ouellette [5] also reported that the influence of the swarm marker (a visually prominent feature over which swarms nucleate) on the swarm morphology decays on a similar scale. Their results provide a strong constraint on how rapidly swarm models must produce collective states. Here I show that the observations of Puckett and Ouellette [5] together with the occurrence of maximal anticorrelated positions [26] are consistent with each individual in the swarm being on average located at a position that is mirror opposite to the average position of all other individuals, so that the average position of the *i*th individual in a swarm containing *N* individuals is given by $\langle x_{i}\rangle =-\frac{1}{N-1}\overset{N}{\underset{\begin{array}{c} j=1\\ j\neq i \end{array}}{\sum }}x_{j}$. With this specification, the least biased (maximum entropy) choice for the distribution of positions is the multivariant Gaussian:

(A11) $$p\left(x_{1},x_{2},x_{3},\ldots x_{N}\right)={\left(2\pi \sigma_{c}^{2}\right)}^{-N/2}exp\left(-\frac{1}{2\sigma_{c}^{2}}\sum_{i=1}^{N}\left(x_{i}-\langle x_{i}\rangle \right)\left(x_{i}-\langle x_{i}\rangle \right)\right)$$

where $\sigma_{c}^{2}$ is a measure of the mean square size of the swarm. This can be rewritten as

(A12) $$p\left(x_{1},x_{2},x_{3},\ldots x_{N}\right)={\left(2\pi \sigma_{c}^{2}\right)}^{-N/2}exp\left(-\frac{1}{2\sigma_{c}^{2}}x_{i}\Lambda_{ij}x_{j}\right)$$

where $\Lambda_{ij}=\frac{N}{N-1}$ if i=j and $\Lambda_{ij}=\frac{3N-4}{{\left(N-1\right)}^{2}}$ if i≠j, and where there is a summation of repeated subscripts. The distribution, Equation (A11), is realizable ($\Lambda_{ij}\;$is positive definite) when N≥3.

Normalized positional covariances are, to good approximation, given by $\sigma_{ij}=1$ if i=j and $\sigma_{ij}=-1/N$ if i≠j, which is indicative of maximal anticorrelated positions.

If the volume of the swarm, $\sigma_{c}^{3}$, is proportional to the population size, *N*, of the swarm, then the volume per individual is predicted to saturate when swarms contain on the order 10 individuals, as observed by Puckett and Ouellette [5]. Puckett and Ouellette [5] found that the approach to the asymptotic state can be accurately represented by a decaying exponential function of the form $V_{ind}=Aexp\left(-\frac{N}{N_{0}}\right)+B$ where the characteristic scale $N_{0}=3.1\pm 0.8$ quantifies the rate of approach with increasing swarm size. Like the observations, model predictions are found to be accurately represented by the decaying exponential function albeit with $N_{0}\approx 1$. But in the presence of a swarm marker of size $\sigma_{s}=1$ (a.u.), the modelling predicts that $N_{0}\approx 5$ (Figure A2). In such cases, the distribution of positions becomes:

(A13) $$p\left(x_{1},x_{2},x_{3},\ldots x_{N}\right)={\left(2\pi \sigma_{c}\sigma_{s}\right)}^{-N}exp\left(-\frac{1}{2\sigma_{c}^{2}}x_{i}\Lambda_{ij}x_{j}\right)exp\left(-\frac{1}{2\sigma_{s}^{2}}x_{i}^{2}\right)$$

Puckett and Ouellette [5] also reported on a related though distinct measure of how the midges arrange themselves in space, namely the average distance from an individual to its nearest neighbour. Puckett and Ouellette [5] found that like the volume per individual, the nearest-neighbour distance falls off rapidly with swarm size for small swarms, but eventually saturates. This behaviour is predicted by stochastic models of the 3-dimensional trajectories of swarming insects, which by construction are exactly consistent with the multivariant distributions of positions, Equation (A11). As observed [5], the characteristic scale, $N_{0}\approx 2$, is larger than for the volume per individual but is less than the observed characteristic scale, $N_{0}=8.6\pm 2.0$. But in the presence of a swarm marker of size $\sigma_{s}=1$ (a.u.), the modelling predicts that $N_{0}\approx 6$ (Figure A2).

Finally, the stochastic modelling predicts that the anticorrelation of spatial positions results in correlated accelerations, as evidenced in an analysis of pre-existing data (Figure A3). Such correlations have until now gone unnoticed, despite numerous attempts to uncover order in the dynamics of swarming insects [2,3,15,27,28].

This analysis leaves open the question as to how the velocity statistics approach the asymptotic regime.
